# The Poses for Equine Research Dataset (PFERD)

**DOI:** 10.1038/s41597-024-03312-1

**Published:** 2024-05-15

**Authors:** Ci Li, Ylva Mellbin, Johanna Krogager, Senya Polikovsky, Martin Holmberg, Nima Ghorbani, Michael J. Black, Hedvig Kjellström, Silvia Zuffi, Elin Hernlund

**Affiliations:** 1 KTH Royal Institute of Technology, Stockholm, Sweden; 2 Swedish University of Agricultural Sciences, Uppsala, Sweden; 3 Max Planck Institute for Intelligent Systems, Tübingen, Germany; 4 Qualisys, Göteborg, Sweden; 5 Sporttotal.tv, Immersive Technologies, Cologne, Germany; 6 CNR Institute for Applied Mathematics and Information Technologies, Milan, Italy

**Keywords:** Scientific data, Biomechanics

## Abstract

Studies of quadruped animal motion help us to identify diseases, understand behavior and unravel the mechanics behind gaits in animals. The horse is likely the best-studied animal in this aspect, but data capture is challenging and time-consuming. Computer vision techniques improve animal motion extraction, but the development relies on reference datasets, which are scarce, not open-access and often provide data from only a few anatomical landmarks. Addressing this data gap, we introduce PFERD, a video and 3D marker motion dataset from horses using a full-body set-up of densely placed over 100 skin-attached markers and synchronized videos from ten camera angles. Five horses of diverse conformations provide data for various motions from basic poses (eg. walking, trotting) to advanced motions (eg. rearing, kicking). We further express the 3D motions with current techniques and a 3D parameterized model, the hSMAL model, establishing a baseline for 3D horse markerless motion capture. PFERD enables advanced biomechanical studies and provides a resource of ground truth data for the methodological development of markerless motion capture.

## Background & Summary

Over the years, capturing and modeling the articulated motion of humans and animals has been a research topic across different disciplines, ranging from medicine^1^ to robotics^2^ to computer graphics^3^. Humans and animals integrate verbal communication with body posture and movements, and understanding body language would greatly advance the design of intelligent interactive artificial systems. Detecting anomalies in articulated motion^4^ can serve as a crucial tool for early health intervention, helping to mitigate potential long-term injuries or disease, hence improving the subject’s quality and length of life. Systems able to synthesize realistic motion can be a useful tool for artists to create characters for games and virtual worlds^5^. Recently, generative AI methods have been wildly trained to synthesize human motion^5–7^.

We focus our study on horses. Horses have historical and cultural significance in most human societies and are one of the oldest domesticated mammals. They have played a significant role in human history, from transportation to warfare, from agriculture to culture, from work to equestrian sports. Their significance is related to their unique bodily strength and speed, made possible by an efficient quadruped locomotor system. This body system has evolved to have bulky muscles close to the upper body, proportionally long limbs that act like pogo-sticks thanks to specialized tendons and a mass reduction of the lower limb and foot that effectively reduces inertia. This optimized locomotor apparatus is however susceptible to injuries as it operates under loading conditions close to its point of failure^8^. Thus, studying the motions of horses has been the focus of researchers in different fields such as robotics^9,10^, biology^11,12^ and veterinary medicine^13,14^.

Marker-based motion capture systems are widely used to capture complex human and animal motions by recording the positions of wearable markers placed on the body in an indoor environment. Marker-based motion capture has demonstrated its significance for motion study^15–17^ and utility across a wide array of applications^14,18^. While marker-based solutions are in need of physical contact with the animal and not scalable to in-the-wild scenarios, computer vision techniques have been developed to implement markerless motion capture, where the articulated motion of a skeleton is inferred from visual data^19–29^. With images and video as the input, straightforward systems provide the image coordinates of skeleton joints as solutions^19–21^. This is not sufficient for many high-quality downstream applications, where a solution independent from the capture geometry is often required. In these cases, a 3D articulated pose, given as the set of 3D rotation angles of skeleton joints, is preferable.

3D markerless motion capture for humans is a novel technology, accurate enough to be used in many applications. In particular, monocular markerless capture, where the 3D pose of the subject is inferred from just one camera, allows designing applications that can exploit low-cost capture devices like smartphones. The basis of these achievements is data-driven methods that leverage large amounts of captured human data. While 2D methods use data in the form of large image datasets with body joint annotations, which are easy to obtain, the data capture task for learning systems outputting 3D poses is significantly more challenging. Moreover, estimating 3D pose from a single view is ambiguous. The problem can be approached by learning explicit 3D pose priors, that constrain the ambiguous solutions to the more likely ones, or learning implicit 3D priors from annotated datasets. The first solution makes use of decoupled data, usually image or video datasets of humans and 2D joints, which are also used for 2D pose estimation, and datasets of human motions. The second solution requires large datasets of images and corresponding 3D poses. These datasets can be obtained at a large scale but only synthetically. Notably, the synthetic dataset generation still requires human articulated motion data. However, relying solely on the priors of human articulated motion is insufficient to achieve markerless motion capture. Seen from the camera, a long leg pointed to the camera can have the same appearance as a shorter leg bent with a different angle. To deal with these ambiguities, a 3D shape prior, encoding the correlation between the body segment proportions, is required.

In the last few years, we have seen a tremendous advance in 3D markerless motion capture for humans. This has been facilitated by the availability of the SMPL (Skinned Multi Person Linear) model^30^, a 3D parametric model, learned from thousands of 3D scans of people, encoding articulated human shape, and the AMASS dataset^31^, a large dataset of human articulated motion, captured with mocap systems, expressed in the parameters space of the SMPL model. Together, SMPL and AMASS incorporate knowledge about how people appear in shape and how they move^7,32–37^.

Animal motion capture has been making strides in recent years but is still behind human motion studies. Analogous to the SMPL model, the SMAL (Skinned Multi Animal Linear) model^38^, learned from 41 toy scans, encodes articulated shapes of quadruped animals. Versions of the SMAL model have been made specifically for dogs^39,40^ and horses^41^. Nonetheless, the data collection on animals is more challenging compared to humans, since it is more difficult to instruct them to perform specific motions and to keep them in fragile indoor environments. Existing animal mocap datasets prefer docile and small animals^42–45^, but are constrained by pose variability and lack the motion diversity that AMASS offers. This results in a paucity of comprehensive animal motion datasets for data-driven motion study, particularly for larger animals, like horses. In the equine veterinary field, motion capture has demonstrated its potential in clinic applications for lameness diagnostics^46,47^. However, within this field, the focus is often limited to capture of locomotion data from a limited number of anatomical landmarks^48–50^ due to difficulty and time constrain in placing markers on the horse’s body. This may lead to less analysis and a lesser understanding of full-body motions.

**Fig. 1:**
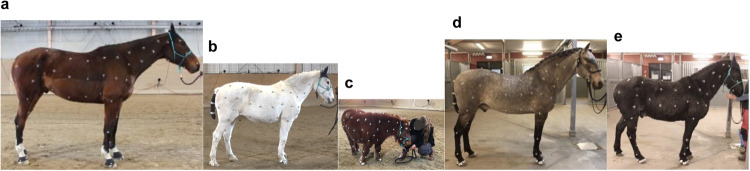
Five horses with different sizes and breeds. (**a**) Horse No. 1. (**b**) Horse No. 2. (**c**) Horse No. 3. (**d**) Horse No. 4. (**e**) Horse No. 5.

To bridge the gap, we introduce PFERD^51^, a dense motion capture dataset of horses of diverse conformation and poses with rich 3D horse articulated motion data. Recorded in an indoor riding arena in Sweden, using an optical motion capture system from the company Qualisys, the dataset includes five horses of different sizes and breeds, to ensure shape diversity (Fig. 1). Over 100 reflective markers were placed on each horse, covering both skeletal structures and soft tissues, to accurately capture motions. The dataset covers a wide variation of horse motions, guided by human instructors, ranging from basic activities like standing, walking, and trotting, to complex motions like the piaffe, the passage, the pirouett, jumping, sitting as shown in Fig. 3. Two highly trained horses perform these advanced motions while the rest of the subjects provide common gaits and motions encountered in every-day horses. Furthermore, to promote the study of markerless motion capture, we provide multiple data types. In line with AMASS, we express 3D horse articulated motion with the hSMAL model^41^. The dataset further enriches its data diversity by including synchronized videos from ten camera views and corresponding 2D joints.

**Fig. 2:**
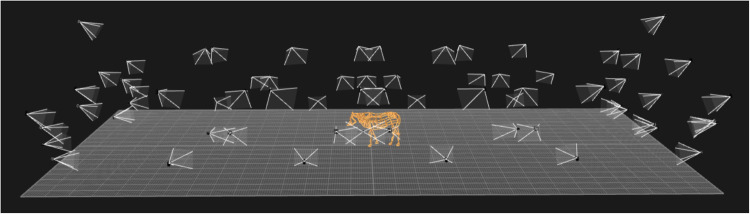
Recording scenario with 56 mocap cameras and ten color cameras using Qualisys system. The system recognizes the markers and the orange lines are connections between the markers.

**Fig. 3:**
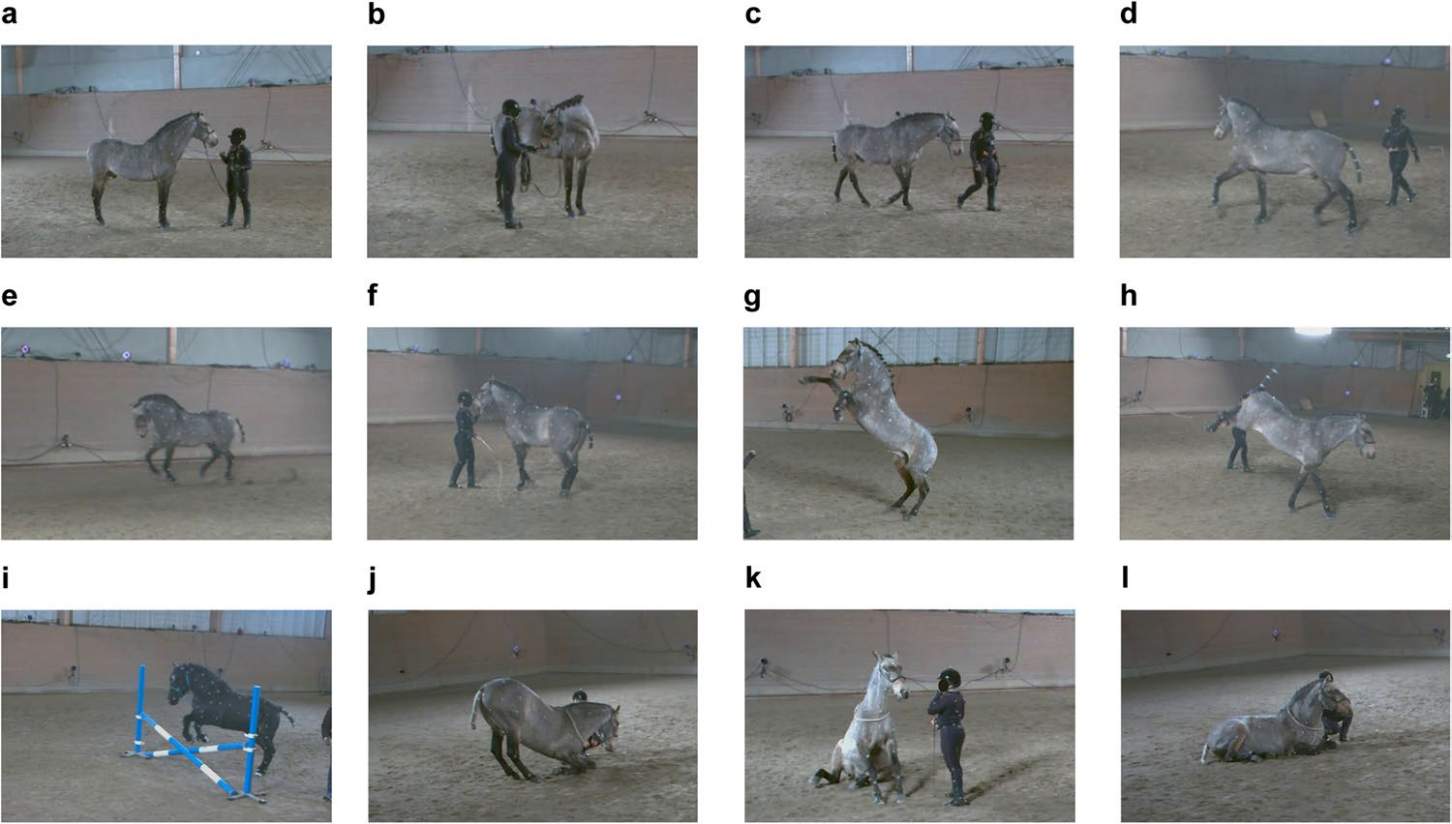
Motion examples. (**a**) Standing. (**b**) Neck bending. (**c**) Walking. (**d**) Trotting. (**e**) Cantering. (**f**) Piaffing. (**g**) Rearing. (**h**) Kicking. (**i**) Jumping. (**j**) Lying down. (**k**) Sitting. (**l**) Lying.

The PFERD dataset serves as an open resource for equine motion research and for the scientific development of computer vision and modeling applications that can benefit horse health and welfare and strengthen our understanding of horse behavior. It provides synchronized 3D data from skin-placed markers and multi-view 2D RGB video streams. The dataset is small in terms of subject numbers, but unique thanks to the wealth of markers placed on the horses’ bodies as well as the subject variation in size, shape, and the rare motions that some of the horses perform. The dataset can be expanded given the detailed descriptions of data capture and model estimation procedures. With this data, we invite researchers to develop both statistical analysis and data-driven methods. We suggest the following tasks:

1. Quantitative motion analysis: The diverse motions and full body marker setup allow for detailed biomechanical studies. The mocap data are, at the time of publication, the most marker-dense horse motion dataset available for research in horses. This data can help veterinary researchers to understand full body motions at a more detailed level than was earlier possible, and it contains unique movements that only highly trained horses can perform.
2. Addressing animal-related computer vision problems: Researchers can utilize this dataset to develop novel computer vision models or refine existing algorithms, promoting the development of markerless motion capture. The 3D motion data can be further used for many graphic tasks, like the development of motion generative models and the improvement of 3D animatable models.
3. Benchmarking in method evaluation through provided groundtruth data: The dataset contains precise 3D mocap data and multi-view RGB video, and can provide both 3D and 2D groundtruth for method evaluation, helping the development of new methods for 3D pose and shape estimation. Furthermore, it enables benchmarking of differences against the state-of-the-art methods we apply here^31,52^.

## Methods

In this section, the procedures used to record the data are explained. In addition, the processing of mocap data and 3D pose data are presented.

### Study subjects

The dataset has a diversity in terms of body shape and motion. We selected five horses of different breeds to provide variable information on shape and size. In terms of diverse motions, all horses performed some basic movements, such as standing (with the head moving from side to side, up and down), moving forward/backward, walking, trotting, and cantering. Two of the horses (Horse No.4 and No.5) performed advanced movements based on signaling ques from their owners, such as pirouetting, rearing, piaffing, kicking, jumping, etc. Table 1 shows the detailed characteristics of the five horses and some motions are listed in Fig. 3.

**Table 1:** Information about the five horses captured in PFERD.

HorseID	Breed	Ages (years)	Gender	Weight (kg)	Wither height (cm)	Marker Num
1	Swedish Warmblood	9	Gelding	702	178	131
2	Irish Pony	22	Gelding	378	130	132
3	Miniaure Shetland Pony	4	Gelding	110	76	120
4	Lusitano	17	Stallion	492	157	132
5	Connemara	17	Gelding	407	142	117

Before each subject was selected, the horse owners were introduced to the aim of data collection and informed about the procedures. Written informed consent was obtained from the owners, permitting the use of the horses’ data for research purposes. The study was non-invasive and the procedure was covered by an animal ethical permission No. 5.8.18-15533/2018. Written consent was provided by all humans appearing in the video recordings.

### Experimental design

In this subsection, the description of the mocap system and marker setup are presented.

#### Motion capture system

The data were collected using Qualisys optical motion capture system on November 26–29, 2020. The system was set up in a riding arena of approximately $19\times 30m$ at the Equine clinic of the University Animal Hospital (UDS) of the Swedish University of Agricultural Sciences (SLU) in Uppsala, Sweden. In total 56 mocap cameras from the Qualisys system (35 Oqus_700 + cameras and 21 Arqus_A12 cameras) and ten RGB full HD video cameras (Miqus_Video cameras) were mounted to the walls of the arena shown in Fig. 2. All cameras were synchronized creating an approximate $16\times 20m$ effective recording volume in the center of the arena. The capture rate of the mocap cameras was 240 Hz and the RGB videos captured by cameras were 20,30,60 Hz, respectively, depending on the data recording.

#### Marker placement and attachment methods

**Fig. 4:**
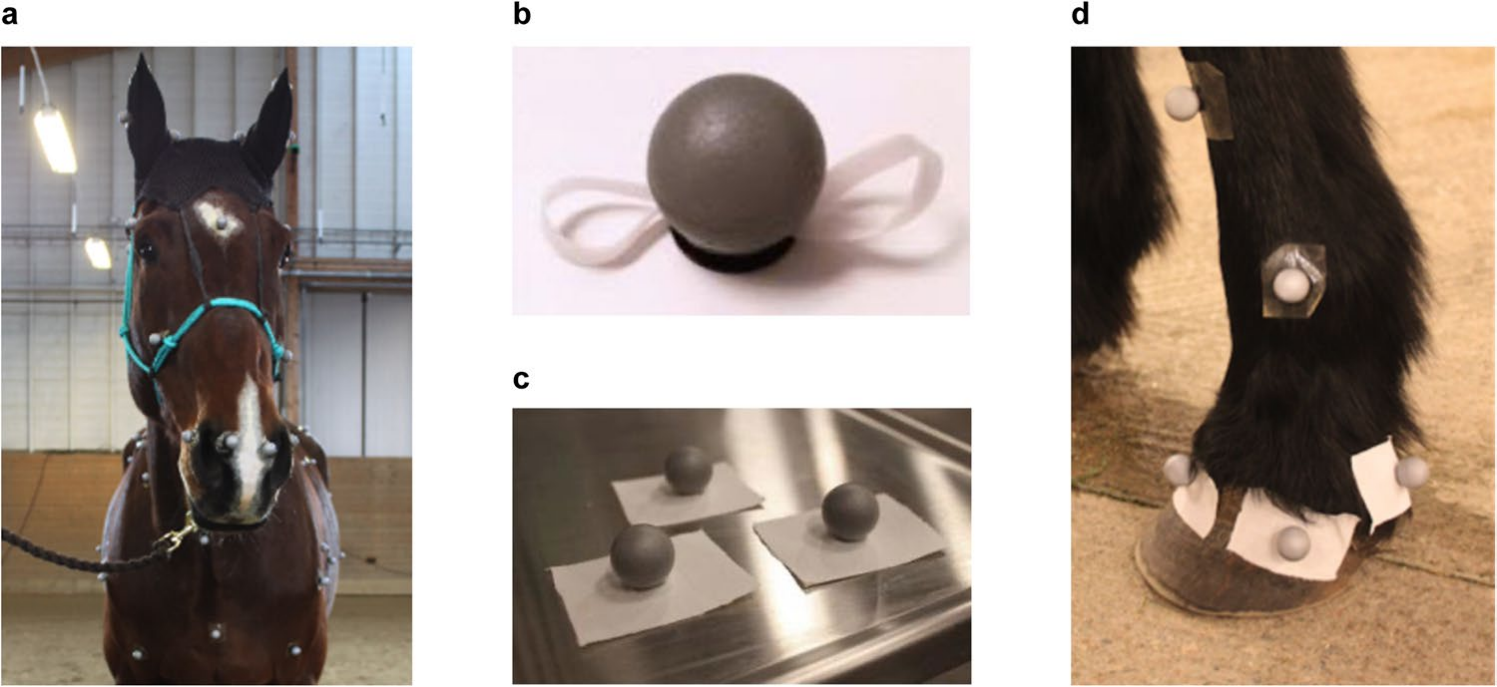
Modified marker attachment methods. (**a**) Placement on the head: Markers on a soft halter and cap. (**b**) Placement on the mane: Markers with incorporated tassels. (**c**) Placement on the hooves: Markers with a piece of fabric in between the basis and marker itself. (**d**) Example of marker placements on the hoof.

Reflective spherical markers with a diameter of 19 mm were attached to the horses’ skin with double-coated adhesive tape cut in pieces of around $2\times 3cm$. Different methods were empirically tested for the more challenging attachment markers to body parts such as the ears and the hooves, shown in Fig. 4.

**Fig. 5:**
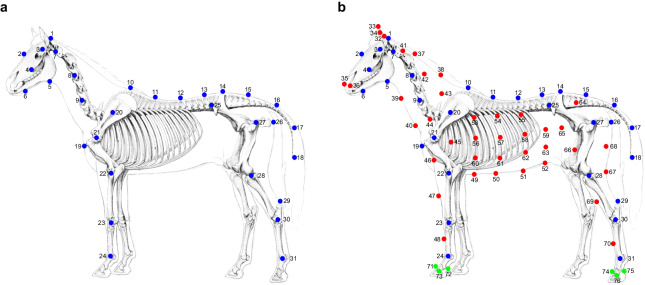
Design marker setup (Modified figure from^55^). (**a**) Skeletal model (in blue), focusing on crucial skeletal segments for locomotion. (**b**) Final model, including skeletal model and markers on soft tissues (in red) and hooves (in green) over the surface.

The marker setup aimed to maximize both captures of body shape and motion and included 132 markers on both skeletal structure and soft tissues. Based on expert anatomical knowledge, markers were separated into three groups. The first group with 50 markers focuses on the precise palpation of anatomical skeletal structures that mark out the most important skeletal segments related to locomotion. Connecting these markers provides a “stick figure”, roughly representing skeletal movement from landmarks on the skin surface. We call these markers the “skeletal model”, see Fig. 5a. The second group of around 70 markers, were dispersed over the horse’s soft tissues, mainly covering the area of the neck, the thoraco-abdominal, and hindquarter segments. The third group of 12 markers were placed in groups of three on each hoof, to allow tracing of rotational motion of the hooves. The final full body marker setup is shown in Fig. 5b. Detailed descriptions are reported in Table 3.

**Table 2:** Detailed data and evaluation metrics for the five horses: includes sequence counts, total data lengths, and frame numbers of silhouette subsets for each horse; evaluations report average 3D distances and average IOU.

Horse ID	SeqNum	Total Length (unit: s)	Average Distance (unit: m)	FrameNum of Silhouette Subset	Average IOU
1	12	887	0.034	17610	0.86
2	9	720	0.032	34878	0.85
3	5	373	0.028	4478	0.79
4	13	1068	0.030	13718	0.86
5	7	420	0.029	6124	0.85
Total	46	3468	0.031	76808	0.85

**Table 3:** Description and placement of marker setup on the horse’s body.

Number	Description	Labels	Landmarks For Marker Placement
1	Poll	Centre	Nuchal crest.
2	Forehead	Centre	Frontal bone between the eyes.
3	Temple	Left/Right	Zygomatic arch.
4	Cheek	Left/Right	Rostral part of facial crest.
5	Jaw	Left/Right	Ventral part at the angle of mandibula.
6	Chin	Left/Right	Ventral part of mandibula at the location of the tooth P2/at the chin groove.
7	Neck_1	Left/Right	Wing of Atlas vertebrae.
8	Neck_2	Left/Right	Transverse process of cervical vertebrae of C3.
9	Neck_3	Left/Right	Transverse process of cervical vertebrae of C5.
10	Back_1	Centre	Dorsal spinal process of thoracic vertebrae of TH5(6) (withers).
11	Back_2	Centre	Dorsal spinal process of thoracic vertebrae of TH12.
12	Back_3	Centre	Dorsal spinal process of thoracic vertebrae of TH18.
13	Back_4	Centre	Dorsal spinal process of lumbar vertebrae of L4.
14	Back_5	Centre	In between the two tuber sacrale.
15	Back_6	Centre	Dorsal spinal process of sacral vertebrae of S5.
16	Tail_1	Centre	Placed at the dorsal spinal process of the caudal vertebrae at the first third part of the distance from Marker15 to Marker18.
17	Tail_2	Centre	Placed at the dorsal spinal process of the caudal vertebrae at the second third part of the distance from Marker15 to Marker18.
18	Tail_3	Centre	Dorsal spinal process of the last of the caudal vertebrae (C15-C21).
19	Chestpoint	Centre	Dorsal part of cartilage of manubrium.
20	ShoulderBlade	Left/Right	Tubercle of spine of scapula.
21	ShoulderJoint	Left/Right	Caudal part of greater tubercle of humerus.
22	ElbowJoint	Left/Right	Lateral collateral ligament over the joint space of the elbow joint.
23	CarpalJoint	Left/Right	Ulnar carpal bone.
24	Foreleg_FetlockJoint	Left/Right	Lateral collateral ligament over the joint space of the fetlock joint on the fore leg.
25	Pelvis_1	Left/Right	Dorsal cranial part of the coxial tuberosity of pelvis.
26	Pelvis_2	Left/Right	Caudal part of the ischiatic tuberosity.
27	HipJoint	Left/Right	Greater trochanter of the femur.
28	StifleJoint	Left/Right	Lateral collateral ligament over the joint space of the stifle joint.
29	PointOfHock	Left/Right	Dorsal part of tuber calcaneus.
30	HockJoint	Left/Right	Tarsal bone IV.
31	Hindleg_FetlockJoint	Left/Right	Lateral collateral ligament over the joint space of the fetlock joint on the hind leg.
32	Ear_Base	Left/Right	Base of the ear.
33	Ear_Top	Left/Right	Top of the ear.
34	Ear_ Side	Left/Right	Lateral side of the middle part of the ear.
35	Nostril_In	Left/Right	Medial wing of the nostril.
36	Nostril_Out	Left/Right	Lateral wing of the nostril.
37	Neck_Top_1	Centre	In the main dorsally on the neck of the first third part from the distance of Marker1 to Marker10.
38	Neck_Top_2	Centre	In the main dorsally on the neck of the second third part from the distance of Marker1 to Marker10.
39	Neck_Bottom_1	Centre	Ventrally on the neck of the first third part from the distance of Marker5 to Marker19.
40	Neck_Bottom_2	Centre	Ventrally on the neck of the second third part from the distance of Marker5 to Marker19.
41	Neck_Up_1	Left/Right	An approximate site in the central part of the area shaped by the markers of 1, 7, 8 and 37.
42	Neck_Up_2	Left/Right	An approximate site in the central part of the area shaped by the markers of 8, 9, 37 and 38.
43	Neck_Up_3	Left/Right	An approximate site in the central part of the area shaped by the markers of 9, 10, 20 and 38.
44	Shoulder_1	Left/Right	An approximate site between the markers of 9, 20 and 21.
45	Shoulder_2	Left/Right	In between the markers of 20 and 22.
46	Chest	Left/Right	At an approximate site in the central part of the area shaped by the markers of 19, 21 and 22.
47	Foreleg_Front_1	Left/Right	Cranial side of the tibia bone in between the markers of 22 and 23.
48	Foreleg_Front_2	Left/Right	Dorsal side of the metacarpal bone III of the foreleg in between the markers of 23 and 24.
49	Belly_1	Centre	Ventral part of the belly at the opposite location of Marker10.
50	Belly_2	Centre	Ventral part of the belly at the opposite location of Marker11.
51	Belly_3	Centre	Ventral part of the belly at the opposite location of Marker12.
52	Belly_4	Centre	Ventral part of the belly at the opposite location of Marker13.
53	Barrel_1	Left/Right	At the first fourth part of the distance from Marker10 to Marker49.
54	Barrel_2	Left/Right	At the first fourth part of the distance from Marker11 to Marker50.
55	Barrel_3	Left/Right	At the first fourth part of the distance from Marker12 to Marker51.
56	Barrel_4	Left/Right	At the second fourth part of the distance from Marker10 to Marker49.
57	Barrel_5	Left/Right	At the second fourth part of the distance from Marker11 to Marker50.
58	Barrel_6	Left/Right	At the second fourth part of the distance from Marker12 to Marker51.
59	Barrel_7	Left/Right	At the second fourth part of the distance from Marker13 to Marker52.
60	Barrel_8	Left/Right	At the third fourth part of the distance from Marker10 to Marker49.
61	Barrel_9	Left/Right	At the third fourth part of the distance from Marker11 to Marker50.
62	Barrel_10	Left/Right	At the third fourth part of the distance from Marker12 to Marker51.
63	Barrel_11	Left/Right	At the third fourth part of the distance from Marker13 to Marker52.
64	Croup_1	Left/Right	At an approximate site in the central part of the area shaped by the markers of 14, 15, 25 and 27.
65	Thigh_1	Left/Right	At the first third part of the distance from Marker25 to Marker28.
66	Thigh_2	Left/Right	At the second third part of the distance from Marker25 to Marker28.
67	Stifle_Back	Left/Right	Caudal side of the leg at the height level of Marker28.
68	Croup_2	Left/Right	In between the markers of 26, 27 and 67.
69	Hindleg_Front_1	Left/Right	Cranial side of the tibia bone in between the markers of 28 and 30.
70	Hindleg_Front_2	Left/Right	Dorsal side of the metacarpal bone III of the hind leg in between the markers of 30 and 31.
71	Hoof_Front_ForeLeg	Left/Right	Dorsal and proximal part of the hoof wall on the front leg.
72	Hoof_Back_ForeLeg	Left/Right	Palmar and proximal part of the hoof bulb on the front leg.
73	Hoof_Side_ForeLeg	Left/Right	Lateral and proximal part of the hoof wall on the front leg.
74	Hoof_Front_Hindleg	Left/Right	Dorsal and proximal part of the hoof wall on the hind leg.
75	Hoof_Back_Hindleg	Left/Right	Plantar and proximal part of the hoof bulb on the hind leg.
76	Hoof_Side_Hindleg	Left/Right	Lateral and proximal part of the hoof wall on the hind leg.

### Data acquisition

In this subsection, the whole procedure of data recording is explained, including mocap system calibration, subject preparation, and type of motion performances recorded.

#### Qualisys calibration

Calibration of the motion capture system was done with wand calibration, according to the manufacturer’s instructions. The video cameras were calibrated along with the marker cameras. For the first calibration, an L-shaped frame with static markers was placed in the approximate center of the capture volume to define the coordinate system. Then a calibration wand with two markers at a fixed distance was moved through the volume to present it to all cameras at different angles. Subsequent calibrations were done with only the calibration wand. The system was recalibrated before recording the first, second and fourth subject.

#### Study subject preparation

**Fig. 6:**
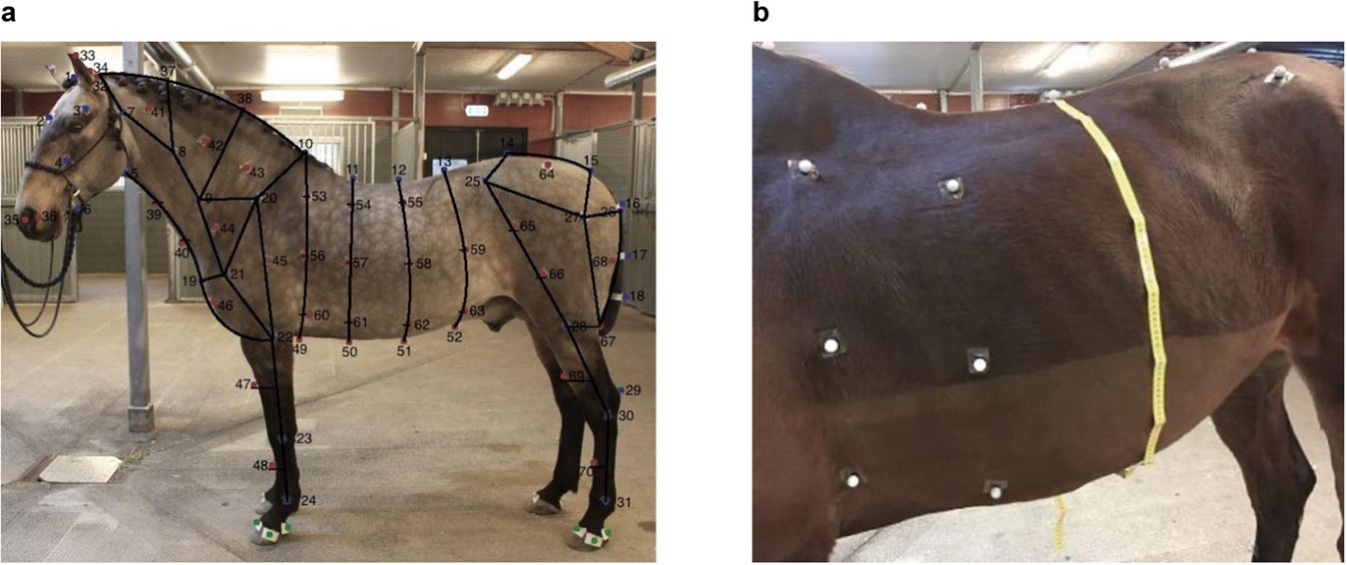
Marker placement measurement on real horses. (**a**) Marker placement and corresponding proportion on the horse’s body. (**b**) A measuring tape was used for marker placement on the barrel.

The fur and the hooves of horses were washed with soap water before attaching markers. Markers were cleaned between different trials if needed. Markers in the skeletal model were placed by palpating specific skeletal structures by two people with anatomical knowledge for precise positioning. The remaining markers were located on the body segment’s proportions using nearby skeletal markers and tape measure as references shown in Fig. 6. Each horse took 2-3 hours to finish all the preparations. The number of markers per horse (shown in Table 1) was a bit different since certain markers had to be excluded for various reasons. For example, Horse No.3 was a small horse, and we had to reduce the number of markers to avoid marker merging, or label-swapping due to them being too close on the small body. Horse No.5 was sweating, resulting in markers not being attached properly, especially markers on the lower belly.

#### Recording sessions

During the recording session for each horse, the horse was first led to the recording arena by the owner and was familiarized with the recording environment. Then, the owner engaged with the horse, using methods such as whistling, waving the whip, and offering treats to guide the horse into performing specific motions. The movements began with fundamental actions, like standing, neck bending, moving forward or backward, as well as, walking, trotting and cantering. The complexity of the movements varied depending on the horse’s ability. For instance, Horse No.4 demonstrated more advanced movements like pirouetting, rearing, piaffing, passage, spanish walk and kicking, while Horse No.5 was rearing and jumping over an obstacle. The dataset comprised different numbers of data sequences for each of the five horses, ranging from 5 to 13, shown in Table 4. While most data recordings lasted approximately one minute, there were exceptions. Each data recording captured more than one motion, allowing for a diverse range of horse movements.

**Table 4:** File lists and motions in the files.

Name	Frame Num	Detected Marker	Motion Description
20201128_ID_1_0001	19200	131	Walking a few steps backward, forward, and side to side.
20201128_ID_1_0002	19200	131	Standing still. Moving head side to side.
20201128_ID_1_0003^1,3^	19200	131	Standing still. Moving head side to side.
20201128_ID_1_0004	19200	131	Walking on straight lines. Walking in curves.
20201128_ID_1_0005	19200	131	Eating from the ground.
20201128_ID_1_0006	19200	131	Walking and trotting in small circles. Walking a few steps backward.
20201128_ID_1_0007	19200	131	Walking, trotting, and canter in small circles.
20201128_ID_1_0008^3^	19200	131	Trotting in straight lines and in curves.
20201128_ID_1_0009	1680	131	Walking, trotting, and cantering in circles.
20201128_ID_1_0010	19200	131	Cantering in circles. Gait transitions.
20201128_ID_1_0011^3^	19200	131	Totting in straight lines. Gait transitions. Walking a few steps backward.
20201128_ID_1_0012^3^	19200	131	Cantering in straight lines. Walking a few steps backward.
20201128_ID_2_0005	19200	132	Walking a few steps backward, forward, and side to side. Moving head side to side and down.
20201128_ID_2_0006	19200	132	Walking in a straight line and with turns.
20201128_ID_2_0007	19200	132	Eating from the ground.
20201128_ID_2_0008	19200	132	Walking with many turns.
20201128_ID_2_0009^3^	19200	132	Trotting and cantering in big circles.
20201128_ID_2_0010	19200	132	Walking and trotting in curves and in big circles.
20201128_ID_2_0011	19200	131	Trotting and cantering in big circles.
20201128_ID_2_0012	19200	132	Trotting and cantering in big circles. Gait transitions. Walking a few steps backward and forwards.
20201128_ID_2_0013	19200	132	Walking and trotting in curves.
20201128_ID_3_0001^2^	32116	116	Walking and trotting in big circles.
20201128_ID_3_0002	14400	116	Walking a few steps backward, forward, and side to side. Moving head up.
20201128_ID_3_0003	14307	117	Moving head side to side. Walking and trotting in circles.
20201128_ID_3_0004	14400	117	Trotting and cantering in circles. Walking a few steps backward.
20201128_ID_3_0005^3^	14400	117	Trotting and cantering in straight lines and circles. Stop from cantering.
20201129_ID_4_0002	19200	130	Walking a few steps backward, forward, and side to side. Moving head side to side.
20201129_ID_4_0003	19200	131	Moving head down and up. Walking.
20201129_ID_4_0005	19200	130	Standing still. Eating from the ground.
20201129_ID_4_0006	55845	132	Standing still. Eating from the ground. Walking and trotting in small circles. Pirouetting.
20201129_ID_4_0007	20663	132	Trotting in small circles around a human. Walking a few steps backward. Pirouetting and rearing.
20201129_ID_4_0008	19200	132	Piaffing/passage (collected trotting). Rearing. Standing still.
20201129_ID_4_0009	14400	131	Piaffing. Rearing.
20201129_ID_4_0010	14400	131	Rearing. Pirouetting. Standing still.
20201129_ID_4_0011	14400	132	Trotting. Kicking with both hindlegs in trotting. Cantering. Rearing. Pirouetting.
20201129_ID_4_0012	14400	129	Spanish walk. Passage. Standing still. Trotting.
20201129_ID_4_0013	14400	130	Pirouetting. Trotting. Kicking with both hindlegs in trotting. Standing still. Cantering.
20201129_ID_4_0019^3^	14400	111	Lying. Standing up. Walking. Lying down.
20201129_ID_4_0020	16799	109	Sitting. Standing up.
20201129_ID_5_0003	14400	116	Walking, trotting and cantering in small circles.
20201129_ID_5_0004^3^	14400	117	Rearing. Walking. Trotting.
20201129_ID_5_0007^3^	14400	116	Rearing. Walking. Trotting.
20201129_ID_5_0008^3^	14400	115	Standing. Moving head side to side, down and up. Walking.
20201129_ID_5_0009^3^	14400	115	Trotting and cantering in circles and in straight lines.
20201129_ID_5_0011^3^	14400	115	Trotting in circles and in straight lines. Walking over the obstacle.
20201129_ID_5_0012^3^	14400	115	Walking. Jumping over an obstacle in trotting.

^1^Later parts of all videos are missing.

^2^Later part of the video from Camera “23348” is broken.

^3^Sometimes markers are out of detection.

### Data processing

In this subsection, we describe the data process from Qualisys and the procedure of learning the 3D model from the mocap data using the hSMAL model and MoSh ++ .

#### Mocap data

The motion capture data was collected using Qualisys Track Manager (QTM) version 2020.3. The collected 2D data were combined into 3D trajectories using the tracking algorithms in QTM, and the trajectories were then labeled. The labeled data has been exported to c3d and fbx format. Since markers might fall off or be occluded during a capture, the number of labeled trajectories might vary slightly for the same horse. In the No.8 trial capture of Horse No.5, some miscommunication with the camera system resulted in a lot of short gaps in the data. Therefore, linear interpolation of the marker position has been used to fill single frame gaps in this one measurement. In the other measurements, no gap fill has been used. The camera calibration information was exported from QTM, including the extrinsic and intrinsic parameters.

#### 2D keypoints and silhouette extraction

For 2D joint extraction, the 3D mocap data was projected onto each image frame from every camera view. This process utilizes the corresponding camera parameters, which are exported from Qualisys, and aligned the first frame of the mocap files to the initial video frame. Considering the differing framerates between the c3d files and videos, a downsample of the c3d files was performed to synchronize the c3d and video frames.

For silhouette extraction, Track Anything^53^, one of the state-of-the-art segmentation models, was employed to extract the 2D silhouettes of each horse in each video frame. The method operated as follows: every five frames within each video, Segment Anything (SAM)^54^ extracted the horse’s mask for the first frame, using the bounding box calculated from 2D key points. Track Anything then applied the results from SAM as a template mask to guide the five-frame segmentation. To ensure the quality of the segmentation, we selected 130 video sequences and manually excluded instances with occlusion or incomplete body visibility.

#### 3D shape and pose modeling

##### The body model

The 3D shape and pose of the horse are modeled and represented through the parameters of the hSMAL body model. As a horse-specific version of SMAL^38^, the hSMAL model^41^ defines a 3D horse mesh, consisting of 1,497 vertices, 2,990 faces, and 36 body segments. hSMAL can be described as a function $\xi (\beta ,\theta ,\gamma )$, where *β* is the shape parameter; *θ* is the 3D pose parameter; *γ* is the model translation. The model is learned from 37 horse toys using the procedure described in^38^. More specifically, a purchased 3D mesh of a horse, created by an artist, is used to create a Global/Local Stitched Shape model (GLoSS)^38^ for horses. The GLoSS model is fitted to each toy scan such that scans have the same mesh topology. To de-correlate body and tail shapes, tails among different toy horses are interchanged to generate a broader range of data. After a process of pose-normalization, the mean template *V*_*mean*_ is computed by averaging the data. The vertex-based residuals between the data and the mean template are modeled by principle component analysis (PCA). *β* represents the coefficients of the learned low dimensional linear space, while *B*_*s*_ defines the shape deformations. More specifically, under the template pose, the shape is given as:

1 $$V={V}_{mean}+{B}_{s}{\beta }^{T}.$$

**Fig. 7:**
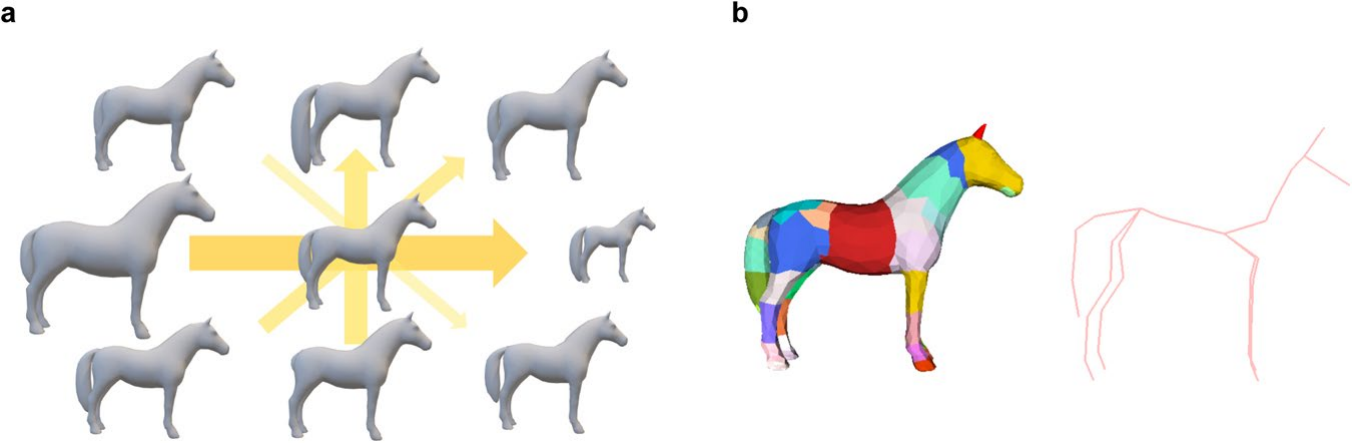
The hSMAL model. (**a**) The hSMAL model and its skeleton. (**b**) The first four principal components in the hSMAL shape space. The arrow width shows from the first to the fourth component. All components are shown with ±2*std*.

The learned shape space of the model is shown in Fig. 7a, where the first three components mainly capture the model’s sizes of the body, the tail, and the neck, respectively. *θ* represents the relative rotation of each joint with respect to its parent joint in the axis-angle representation according to the skeleton tree defined in the model. The *θ* parameter is a vector of dimension $3\times 36=108$. The skeleton joint positions are manually defined (Fig. 7b) to better represent the animal anatomy similar to^45^. The final mesh is then posed with Linear Blend Skinning (LBS)^30,38^ and shifted with translation parameter *γ*. More details are in the original papers^38,41^. We used the first 10 PCA coefficients of the shape space as the shape parameters.

##### Model fitting

The parameters of a 3D articulated shape model can be estimated from mocap markers using the MoSh^52^ and MoSh++^31^ methods. These methods consider the fact that markers cannot be attached to fixed positions on the human body, especially when the human is moving or the markers are on soft tissues and solve not only for model parameters, but also for the marker position on the body surface. MoSh++, the updated version of MoSh, has been applied to different human mocap datasets to create a unified dataset, AMASS, which includes markers and aligned SMPL model parameters.

Following MoSh++^31^, we use two stages to capture the 3D shape and pose of the horse using the hSMAL model from mocap data. We use a similar notation as in MoSh++. Please check for more details in the original paper^31,52^.

Stage I: Stage I focuses on estimating the shape and marker positions. A marker parameterization denoted as $m({\widetilde{m}}_{i},\beta ,{\theta }_{t},{\gamma }_{t})$ is utilized to estimate marker positions considering the body’s shape, pose and location. More specifically, the latent markers ${\widetilde{m}}_{i}$ are mapped to the world by accounting for the model parameters $(\beta ,{\theta }_{t},{\gamma }_{t})$ at a particular frame *t* for marker *i*. To do this, *F* frames are randomly selected from subject-specific mocap sequences. The goal is to optimize the model parameters ($\beta$, $\Theta =\{{\theta }_{1:F}\}$, $\Gamma =\{{\gamma }_{1:F}\}$) and latent marker positions $\widetilde{M}=\{{\widetilde{m}}_{i}\}$, based on the observed marker positions $M={\{{m}_{i,t}\in {M}_{t}\}}_{1:F}$. More specifically, an objective function is defined as:

2 $$E\left(\widetilde{M},\beta ,\Theta ,\Gamma \right)={\lambda }_{D}{E}_{D}\left.\left(\widetilde{M},\beta ,\Theta ,\Gamma \right)\right)+{\lambda }_{R}{E}_{R}\left(\widetilde{M},\beta \right)+{\lambda }_{I}{E}_{I}\left(\widetilde{M},\beta \right)+{\lambda }_{\beta }{E}_{\beta }\left(\beta \right)+{\lambda }_{\theta }{E}_{\theta }\left(\theta \right)$$

where ${E}_{D}$ measures the distance between the parameterized markers $m({\widetilde{m}}_{i},\beta ,{\theta }_{t},{\gamma }_{t})$ and the observed markers ${m}_{i,t}$, ${E}_{R}$ ensures the markers are at an appropriate distance from the model surface (here we set 10 mm). ${E}_{I}$ maintains the parameterized markers close to their initial positions. ${E}_{\beta }$ and ${E}_{\Theta }$ are regularizers related to shape and pose prior to the hSMAL model defined in^41^. Finally, a four-staged approach is performed to help avoid getting stuck in local optima. Here we randomly selected $F=12$ frames from sequences where the horse was in more static poses, as it allowed for better optimization. It was worth noting that some markers may not be visible in these selected sequences. We used specific values for ${\lambda }_{D}=105.0\times d$, ${\lambda }_{R}=10300.0$, ${\lambda }_{I}=250.0$, ${\lambda }_{\beta }=14.5$, ${\lambda }_{\theta }=7.5$ and a scaling factor $d=50/n$, to deal with varying numbers of markers, where 50 was the marker number of the skeletal model and *n* was the observed mocap marker number in a frame.

Stage II: Stage II focuses on optimizing the 3D poses from all subject-based sequences. The body shape *β* and latent marker positions of each subject in Stage I are determined and kept fixed during Stage II. More specifically, we minimize:

3 $$E(\theta ,\gamma )={\lambda }_{D}{E}_{D}(\theta ,\gamma )+{\lambda }_{\theta }{E}_{\theta }(\theta )+{\lambda }_{u}{E}_{u}(\theta )$$

where ${E}_{D}$ and ${E}_{\theta }$ are the same as in Stage I, measuring the alignment of the model with the observed data and maintaining specific constraints. *E*_*u*_ is a temporal smooth term, ensuring the 3D poses changed over time are natural and smooth. Here we set ${\lambda }_{D}=480.0\times d$, ${\lambda }_{\theta }=2.3\times q$, ${\lambda }_{u}=2.5$. A variable $q=1+\left(\frac{x}{|M|},\ast ,2.5\right)$ was introduced based on the number of missing markers *x*. The more markers were missing, the higher the pose constrained in every given frame.

##### Hyper-parameter search

Certain hyperparameters *λ* during the two-stage optimizations were determined by line search on a synthetic dataset, as inspired by MoSh++. The synthetic dataset was first created using the toy shapes from the training data of the hSMAL model. We placed 38 synthetic markers on the model with toy shapes and animated the model. We divided this data into a training set, consisting of 32 toys and five animations and a validation set with five toys and two animations.

The searching process was adjusting one parameter while keeping other parameters fixed, both in Stage I and Stage II fitting, using different random seeds on training and validation datasets. In Stage I, we initialized the marker positions by randomly placing them near the true position. The goal was to find a better combination of $({\lambda }_{D},{\lambda }_{R},{\lambda }_{I},{\lambda }_{\beta },{\lambda }_{\theta })$ in Eq. 2. We aimed to find the values that provide the relatively lower distance between the estimated markers and the synthetic true markers within the selected 12 frames in both training and validation sets. In Stage II, the process was to find a better combination of $({\lambda }_{D},{\lambda }_{\theta },{\lambda }_{u})$ in Eq. 3. Here we aimed to minimize the error on all vertices of the model between the estimated results and the true results in both the training and validation set.

## Data Records

The datasets are available at Harvard Dataverse^51^. The data is organized by subject folder with [Subject ID]. The name of files for each trial starts with *[Record Date]_[Subject ID]_[Trial Number]*, indicated as *[Trial Name]*. Each subject folder stores six sub-folders with complete trials:

- C3D_DATA: One C3D file per trial, exported from the QTM software, referenced as *[Trial Name].c3d*.
- FBX_DATA: One FBX per trial, with the whole scenario including all information of cameras and 3D position of the subject, exported from the QTM software, referenced as *[Trial Name].fbx*.
- VIDEO_DATA: Each folder per trial named as *[Trial Name]*, with videos from ten camera views, references as *[Trial Name]_[Camera Code].avi*.
- SEGMENT_DATA: Selected segmentation subsets for evaluating the fitting results, referenced as *[Trial Name]_[Camera Code]_seg.mp4*.
- MODEL_DATA: NPZ files, referenced as *[Trial Name]_hsmal.npz* includes the hSMAL parameters per trial. Another NPZ file, reference as *[User ID]_stagei.npz* contains the latent representation of the markers.
- KP2D_DATA: Each folder per trial named as *[Trial Name]*, with 2D keypoints projected from 3D mocap data into video frames with ten camera views, referenced as *[Trial Name]_[Camera Code]_2Dkp.npz*.
- CAM_DATA: The camera parameters from ten camera views, referenced as *Camera_Miqus_Video_[Cam ID].npz*.

The correspondences are as follows:

[Record Date]: Recording date (e.g. 20201128 or 20201129)

[Subject ID]: Subject ID (e.g. ID_1, ID_2, ID_3, ID_4, ID_5)

[Trial Number]: Trial number (e.g. 0001, shown in Table 4)

[Camera Code]: Camera code (e.g. Miqus_65_20715)

[Camera ID]: Camera ID (e.g. 20715, 21386, 23348, 23350, 23414, 23415, 23416, 23417, 23603, 23604)

## Technical Validation

In this section, we first provide calibration errors from QTM software and quantitative and qualitative results of the reconstructed 3D model.

### Mocap data

The motion capture system was calibrated three times over the two days of data capture. The standard deviation of the wand length varies from 1.0–1.9 mm for the calibrations, with an average camera residual between 2.6–2.9 mm during calibration. For the different captures, the average camera residual varied between 1.5–3.1, with higher residuals when the horse moves close to the edge of the covered volume.

### 3D model evaluation

#### Qualitative Results

**Fig. 8:**
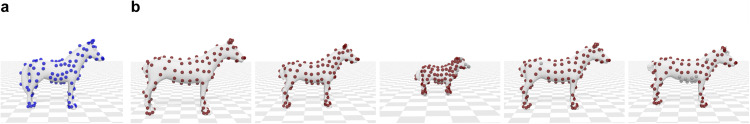
Optimized shape and markers. (**a**) Initial guess of markers (in blue) on the template shape. (**b**) Optimized shape and optimized marker locations (in red) of five horses. Markers (in gray) are non-observable labels in observed frames in Stage I.

We show the optimized shape and marker positions in Fig. 8. The positions of the markers (in red) have been optimized to fit different shapes, starting from their initial guess positions (in blue) on the template shape. Additionally, some markers (in gray) represent markers not visible during Stage I, which could be either the exclusion of attaching certain markers or fail marker detection during the data recording process.

**Fig. 9:**
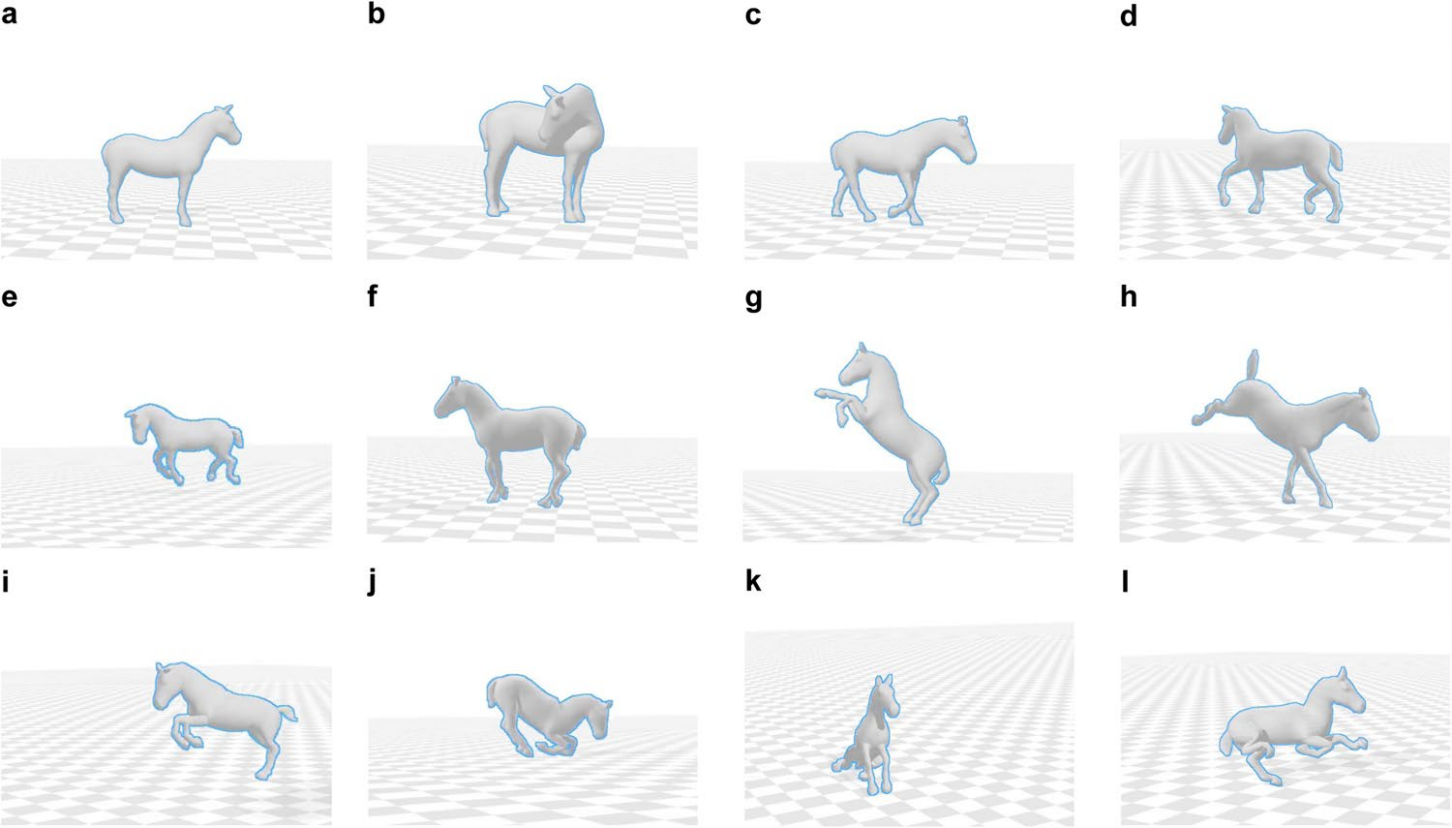
Visualization of different motions. (**a**) Standing. (**b**) Neck bending. (**c**) Walking. (**d**) Trotting. (**e**) Cantering. (**f**) Piaffing. (**g**) Rearing. (**h**) Kicking. (**i**) Jumping. (**j**) Lying down. (**k**) Sitting. (**l**) Lying.

Examples of the captured 3D model are displayed in Fig. 9 with corresponding motions illustrated in Fig. 3. The 3D shape and pose representations, derived from mocap data, effectively capture the horse’s real motions, even in challenging poses, like prancing and kicking. However, room for improvement remains in capturing more complex poses, such as sitting and lying down, especially when the limbs are in unusual positions.

**Fig. 10:**
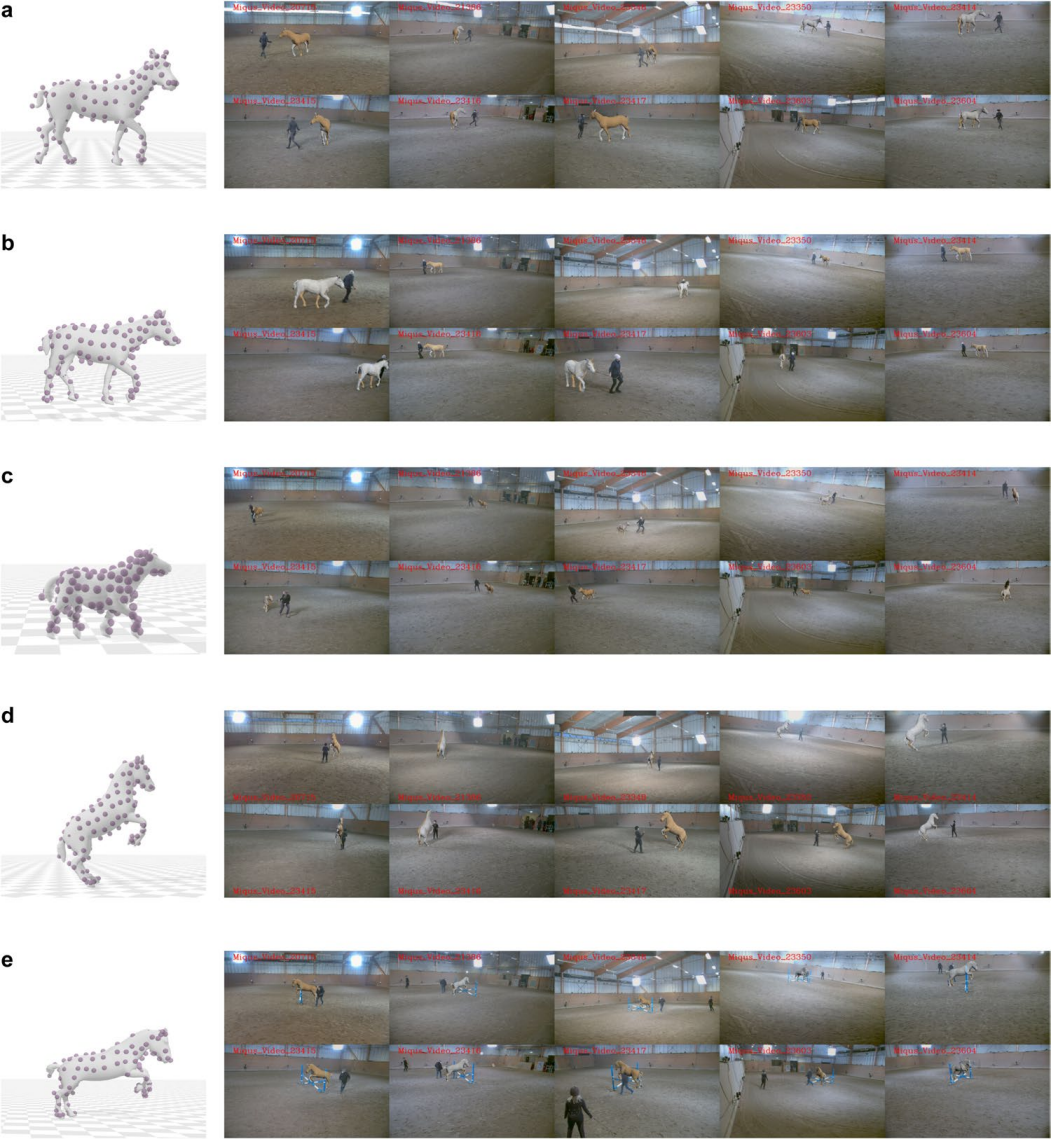
Visualization of example frames in different horses. Left: The 3D model and the corresponding mocap data (purple). Right: Project the 3D model in the same image frame in ten views. Left body (in yellow) and right body (in lightgray). (**a**) Horse No. 1. (**b**) Horse No. 2. (**c**) Horse No. 3. (**d**) Horse No. 4. (**e**) Horse No. 5.

Figure 10 provides results for the five horses. The images on the left side display the fitting results and the mocap frame, while the images on the right side provide a view of the reconstructed model as seen from ten different camera views. This visual comparison highlights the precision of the captured 3D shape and pose of the horses.

#### Shape Visualization

We provide the visualization of the estimated 3D horse shapes. Figure 12b shows the UMAP visualization of the components of the shape parameters from our five subjects, together with those of all the hSMAL toy training data. We can observe that Horse No. 3, the smallest pony, is quite distinct from the other four horses (Fig. 12a).

**Fig. 11:**
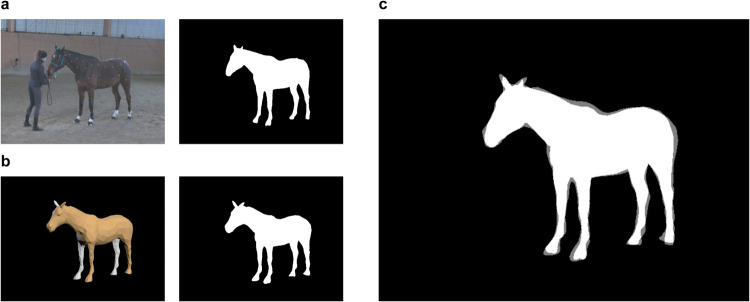
3D model evaluation with intersection over union (IOU). (**a**) The video frame and the silhouette extracted from video. (**b**) The image and the silhouette obtained from projecting the model into the image plane. (**c**) The overlap of the two silhouettes.

**Fig. 12:**
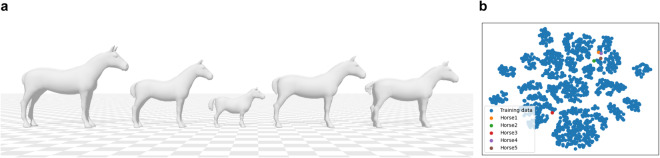
Shape visualization of the five PFERD horses and UMAP analysis with the first two components of the shape parameters of these five horses and all the training toy data. (**a**) The shape of the PFERD horses as reconstructed in hSMAL. (**b**) UMAP visualization.

#### Quantitative Results

##### 3D Mocap Error

 To evaluate the accuracy of the model in capturing the shape and pose information from the mocap data, we analyze the Euclidean distance between the observed markers and the estimated virtual markers in each frame. As failed markers and noisy labels are inevitable, we focus on frames where more than 23 markers are detected. The results, detailed in Table 2, show results per horse across all trials and the average between different horse subjects is 0.031 meters.

##### 2D Silhouette Error

We measure the accuracy of the captured 3D shape and pose by calculating the intersection over union (IOU) (Eq. 4) to measure the overlapping (Fig. 11c) between the extracted segmentation $S$ (Fig. 11a) and the silhouette $\widetilde{S}$ (Fig. 11b), obtained by projecting the corresponding 3D model with the camera. We calculate the IOU of frames that are inside the selected silhouette subsets with more than 23 markers detected. Table 2 shows results over the selected silhouette subset per horse, with an average IOU of 0.85 across different horse subjects. Intersection over union is defined as:

4 $$IOU=\frac{\left|S\cap \widetilde{S}\right|}{\left|S\cup \widetilde{S}\right|}$$

#### Current limitations

**Fig. 13:**
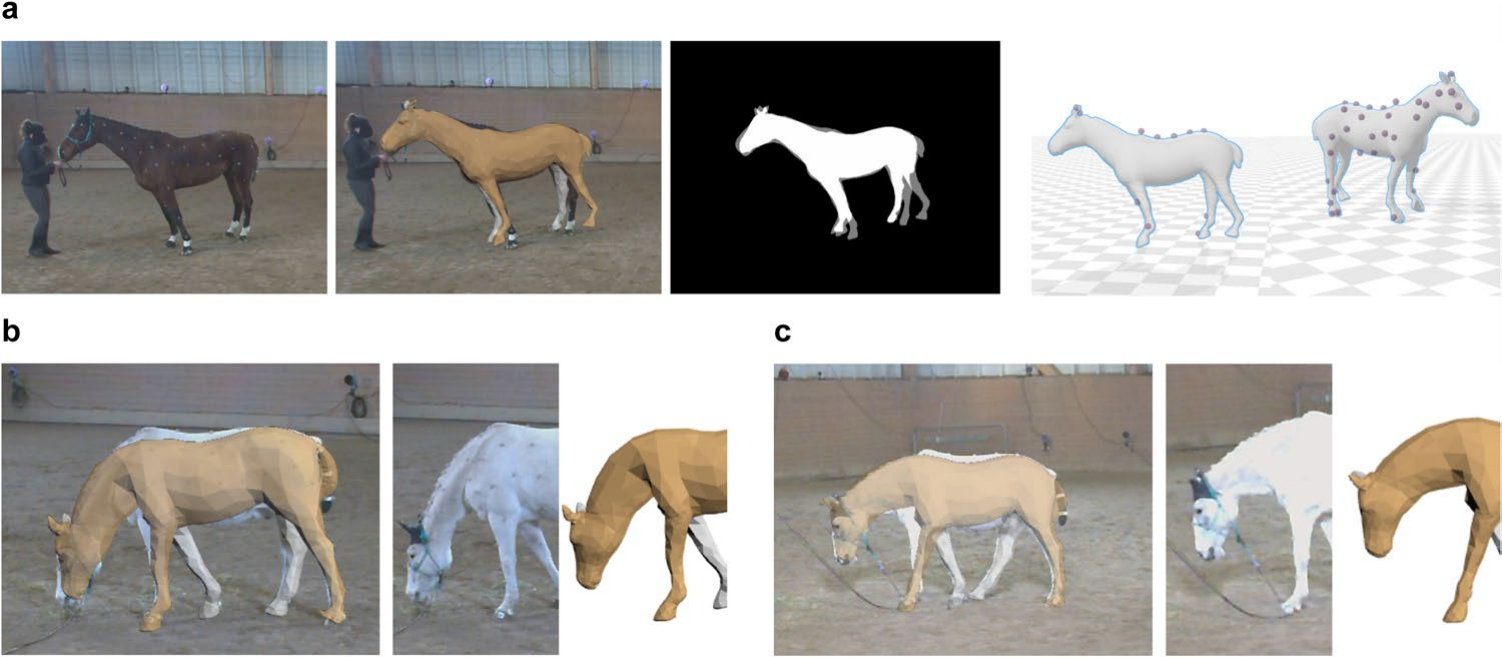
Imperfect capture. (**a**) Missing mocap markers. From left to right: original video frame, the overlapping model with the image, overlapping silhouette, visualization of the model and mocap markers in two different views. (**b**) and (**c**) are two examples of model limitations in real-world representation. Example 1 (b), where the model did not capture the shape of the neck and back well. Example 2 (c), where the model did not capture the shape of the neck and the shoulder well.

Our current dataset has some limitations, as in Fig. 13, where the motion is not well captured. While the mocap system is precise, occasional irregularities may appear. For example, Fig. 13a shows a case where some of the markers are undetected, leading to misalignment of the model with the silhouette. Another limitation is the hSMAL model itself, which is learned from toy scans, and cannot perfectly represent real-world horses. As seen in Fig. 13b,c, certain body parts like the shape of the cheek and the back cannot be accurately captured, indicating the need for a more precise model.

## Usage Notes

The provided FBX files can be imported into different animation applications, including Blender and any other application that supports loading fbx files. The provided c3d files can be processed using Python.

Researchers should be aware of certain anomalies in the data. Despite our meticulous data collection, a small number of irregularities may appear as we mentioned in the previous section, including missing markers, swapped marker labels, and noisy marker positions. This results in weird poses of the reconstructed model when too few correct markers are visible within a given frame. To mitigate this, we set a threshold requiring more than 23 visible markers for pose evaluation. However, these potential issues may influence the final results.

The silhouettes generated through deep learning methods are not fully infallible but can be considered as pseudo-ground truth. Even when we carefully select a subset manually comprised of relatively complete silhouettes, it’s important to note that potential errors remain.

Additionally, discrepancies in response times among different camera sensors exist, despite all cameras being synchronized. In our current setup, we align the first frame of the mocap files with the first video frame of the corresponding videos, which may still result in minor inaccuracies. Users should be cognizant of this small margin of error during data analysis and interpretation.

## Data Availability

We make available functions for users to use our datasets: • Loading c3d files and the hSMAL model with the captured parameters to visualize the mocap data and the fitted results. • Projecting the reconstructed model in image planes with provided camera information. • Quantitative evaluation using the mocap data and silhouette subsets. Further detail about environment settings and code usage can be found in https://github.com/Celiali/PFERD.git.
